# Collection of Hairy Roots as a Basis for Fundamental and Applied Research

**DOI:** 10.3390/molecules27228040

**Published:** 2022-11-19

**Authors:** Anna Yurievna Stepanova, Maria Viktorovna Malunova, Evgeny Aleksandrovich Gladkov, Sergey Viktorovich Evsyukov, Dmitry Viktorovich Tereshonok, Aleksandra Ivanovna Solov’eva

**Affiliations:** K.A. Timiryazev Institute of Plant Physiology, Russian Academy of Sciences, 127276 Moscow, Russia

**Keywords:** hairy roots, secondary metabolites, collection

## Abstract

Due to population growth, instability of climatic conditions, and reduction of the areas of natural ecosystems, it becomes necessary to involve modern biotechnological approaches to obtain highly productive plant material. This statement applies both to the creation of plant varieties and the production of new pharmaceutical raw materials. Genetic transformation of valuable medicinal plants using *Agrobacterium rhizogenes* ensures the production of stable and rapidly growing *hairy roots* cultures that have a number of advantages compared with cell culture and, above all, can synthesize root-specific substances at the level of the roots of the intact plant. In this regard, special attention should be paid to the collection of hairy roots of the Institute of Plant Physiology RAS, Russian Academy of Sciences, the founder of which was Dr. Kuzovkina I.N. Currently, the collection contains 38 hairy roots lines of valuable medicinal and forage plants. The review discusses the prospects of creating a hairy roots collection as a basis for fundamental research and commercial purposes.

## 1. Introduction

Plants produce a wide range of substances useful to mankind which are used in the food industry as biologically active additives, feed additives, medicines, as well as flavorings and food colorings [1,2,3,4]. Approximately 25% of the world pharmaceutical market is products obtained from plants [4]. However, often high-value secondary metabolites are synthesized by plants in small amounts under natural conditions. For example, about 10,000 kg of dry bark of *Taxus* sp. is required to obtain 1 kg of taxol [5]. Taxol (paclitaxel) is the first drug from the taxan group, which entered clinical practice and firmly took the leading position in the treatment of the most frequent malignant tumors—breast cancer, ovarian cancer, and non-small-cell lung cancer [6,7,8,9]. In addition, Taxol has shown promising results in the treatment of Kaposi sarcoma [10]. Taxol is also being studied for non-cancerous diseases that require microtubule stabilization to avoid cell proliferation and angiogenesis, such as psoriasis, and for the treatment of Alzheimer’s or Parkinson’s disease [11,12]. Taxol’s annual turnover by 2000 was $1.5 billion [13]. To obtain 1 kg of vinblastine and 1 g of vincristine, which are also widely used anticancer drugs, 530 kg of fresh *Catharanthus roseus* (L.) G. Don. leaves is needed [14]. Vinblastine and vincristine are recommended for the treatment of rapidly proliferating neoplasms (hematosarcoma, myeloma, acute leukemia, etc.), breast cancer, neuroblastoma, chorionepithelioma, and lymphogranulomatosis [15].

Due to the global problem of instability of climatic conditions and the shortage of plant raw materials, it is necessary to use biotechnological approaches to obtain it in sufficient quantities, namely, the cultivation of plant cells and plant organs in vitro. In this regard, special attention is drawn to biotechnological approaches associated with the cultivation of cultures in vitro: undifferentiated—callus and cell suspension cultures and differentiated—adventitious and hairy roots.

The biotechnology of higher plants developed in the middle of the last century. Biotechnology in most cases is understood as an application of callus and suspension cultures, which, as a rule, are combined under the general name “tissue culture”. The first patent for the production of substances using plant tissue culture was obtained in 1956 [16]. Even today, the greatest number of publications is devoted to the direction associated with the use of in vitro cultured cells, namely, undifferentiated cultures. Numerous reviews discuss the production of metabolites important for humans from suspension and callus cultures [1,17,18,19]. Some authors have noted that the content of secondary metabolites in the resulting suspension and callus cultures was higher than in intact plants [4,19,20]. However, the opposite result was obtained in most cases [21,22,23,24,25,26]. Difficulties arise during large-scale cultivation due to the instability of the synthesis of substances by undifferentiated cultures in a liquid medium [19,27]. In addition, the synthesis of some pharmacologically important substances may not be possible in undifferentiated cultures [17,27]. In this regard, attention is drawn to differentiated cultures: shoots, adventitious, and hairy roots (transformed roots). Adventitious roots are induced on media with high auxin and low cytokinin content. However, despite the studies that showed the promise of using adventitious [28,29,30] (untransformed) roots, most studies noted the disadvantages of their use, primarily slow growth and termination of the synthesis of target substances [27,31]. On the contrary, hairy roots have rapid growth on a hormone-free medium and a high and stable synthesis of essential substances, which has been repeatedly shown [31,32,33,34,35,36,37,38]. Therefore, the technology of hairy roots is very promising.

In our group of specialized root metabolism at the Institute of Plant Physiology, the first studies were carried out with non-transformed adventitious roots, but with the appearance of the possibility of obtaining hairy roots, these studies were discontinued [39]. Thus, the technology of hairy roots in Russia, as well as in other countries, arose later than cellular biotechnology and the culture of isolated roots. Nevertheless, the number of works related to hairy roots is steadily increasing.

This review discusses obtaining hairy roots as producers of valuable metabolites, as well as the prospects of creating a collection of hairy roots consisting of different types of crops for fundamental and commercial purposes.

## 2. The History of the Development of the Hairy Roots Trend in the World

The history of the emergence of hairy roots as an object of scientific research began in the late 19th to early 20th century and is associated primarily with the American phytopathologist Smith, who studied the formation of crown galls and hairy roots in a number of fruit plants [40]. In November 1908, cultures of bacteria capable of infecting new plants were isolated from the hairy roots of an apple tree, which is described in detail in a large-scale 215-page work by American authors, accompanied by many high-quality photos to document the experimental material obtained [40]. The same paper describes numerous experiments in which bacteria isolated from one plant species successfully infected another and formed similar hairy roots in that one. However, the original causes of the modification of plant organisms under the influence of crown gall bacteria in some and hairy roots in others remained unclear.

The first work that showed that the formation of hairy roots is caused by the transfer of genetic material from *Agrobacterium rhizogenes* dates back to 1982 [41]. *Agrobacterium rhizogenes* is a Gram-negative bacillus, a symbiotic bacterium that currently has been renamed (also named *Rhizobium rhizogenes*). The article drew a parallel between two types of pathogenic bacteria—*Agrobacterium tumefaciens* and *Agrobacterium rhizogenes* and concluded that the Ri-plasmid of *A. rhizogenes*, as well as the Ti-plasmid of A. tumefaciens, can be a vector for the transfer of genetic material. Starting from this date, researchers began to consider hairy roots not just as a neoplasm resulting from the attack of a pathogenic bacterium, but also as a promising model for studying the features of secondary metabolism and, ultimately, as producers of natural products.

In 1997, the monograph “Hairy Roots: Culture and Applications” (Doran P.M., Harwood Academic) was published on the status of research activities in the field of hairy root biotechnology at that time [42]. The monograph outlined laboratory protocols for the initiation and cultivation of hairy roots; described the use of hairy roots as producers of secondary metabolites; as an expression system for the production of antibodies; considerations are given for their large-scale cultivation in bioreactors. Studies on phytoremediation were also presented. The monograph combines the work of scientists from many fields, from genetics and molecular biology to horticulture, medicine, environmental research, and biotechnology.

According to 2006 data since the time of the first publications, the roots of more than 140 plant species belonging to 40 families have been introduced into in vitro culture using methods of genetic transformation of roots [43]. The number of introduced species is only presently increasing [34]. However, hairy roots are mainly induced in dicotyledonous plants. Obtaining hairy roots in monocotyledonous plants is difficult since the infection of such plants with *A. rhizogenes* is a very rare event in nature. One of the reasons for this may be the lack of production of wounder phenolic compounds, such as acetosyringone [44]. It acts as a chemotactic agent at very low concentrations and activates *vir*-genes on Ri-plasmids, which initiate the infection process for the transfer of T-DNA [45,46]. Nevertheless, there are works on obtaining hairy roots in representatives of the monocotyledonous class, such as *Alstroemeria* [47], *Chlorophytum borivilianum* [48], *Zea mays* [49], *Crocus sativus* [44], etc. These works are of great interest since a significant number of medicinal plants are monocotyledonous. One of the ways to overcome the difficulty of agrobacterial transformation for monocotyledonous plants is to use the microparticle bombardment method, since there is no limitation to the range of hosts with this method. It was developed in 1990 by Sanford and coworkers [50]. Indeed, in some cases, the microparticle bombardment method has been successful enough to produce hairy roots in both monocotyledonous and dicotyledonous plants [51,52,53]. Despite this, most authors tend to use Agrobacterium even in the case of monocotyledonous plants, since this method is simpler and does not require expensive equipment. The advantage of agrobacterial transformation over the microparticle bombardment method is the integration of one copy of the transgene in most cases, the low incident of transgene silencing, and the ability for long DNA segment transfer [47,52,54].

For 40 years since the publication of the first works, hairy roots have been used as producers of secondary metabolites, such as alkaloids, anthocyanins [37,55], flavonoids, ginsenosides, stilbenes, lignans, terpenoids, and shikonin [56]; as well as recombinant proteins such as vaccine [57,58], monoclonal antibodies [59], and therapeutic proteins [60]. Studies on the possibility of using hairy roots in phytoremediation are being conducted [61,62]. However, to the greatest extent, the hairy roots are used as a source of pharmacologically valuable secondary metabolites. It should be noted that, in most cases, the content of medicinal substances in hairy roots is at the level or higher than their content in the roots of intact plants (Table 1). One of the reasons for the high synthesis of various secondary metabolites is the presence of *rol*-genes, primarily *rol*B and *rol*C [63,64]. Bulgakov et al. showed that *rol*B affects the expression patterns of MYB factors controlling the early steps of flavonoid biosynthesis [64].

Hairy roots can synthesize a number of secondary metabolites that are not typical for the intact roots of plants [65]. It has been shown that naphthochinon lawson, which accumulated in the aerial parts of *Lawsonia inermis* L., was not found in plant roots and adventitious roots cultivated in vitro, but was presented in hairy roots [66]. It was considered that only artemisin accumulates in the aboveground part of the *Artemisia annua* plant [67], but it was shown later that hairy roots can also produce artemisin [68]. 

In the case of an insufficient level of synthesis of secondary metabolites in root cultures, either elicitors or genetic engineering methods are used to increase their content [69]. The use of elicitors is well highlighted in a recently published review covering the period from 2010–2022 [21]. According to this review, methyl jasmonate acts as the main elicitor and the content of secondary metabolites in the hairy roots of various families was significantly increased with it, but the greatest effect was shown in representatives of the genus *Lamiaceae* [21]. 

The introduction of genes controlling the synthesis of valuable secondary metabolites makes it possible to obtain hairy roots with a stable synthesis of substances regardless of the action of external factors. For example, the introduction of transcription factors *WRKY1,2* [70,71], *MYB98* [72], key genes involved in the tanshinone biosynthetic pathway *HMGH* and *DXR* [73], *GGPPS* and *DXSII* [20] increased the synthesis of tanshinones in the hairy roots of *Salvia miltiorrhiza* by 1.4–21 times [74]; overexpressing CrORCA4 in *Catharanthus roseus* increased tabersonine synthesis by 40 times [75]. The introduction of the maize transcription factor *ZmLc* and *Arabidopsis* transcription factor *AtPAP1* made it possible to increase the content of three main flavones (baicalin, baicalein, and wogonin) in hairy roots of *Scutellaria baicalensis* by 322% and 532%, respectively, by a comprehensively upregulating flavonoid biosynthesis of pathway genes [76]. Through the introduction of curcumin biosynthetic pathway genes, it was possible to increase the level of curcumin and its glycosides in *Atropa belladonna* hairy roots [77]. The number of such works is steadily increasing.

All of the aforementioned makes hairy roots technology a powerful tool for both fundamental and applied research.

**Table 1:** The benefits of using hairy roots. The examples of studies in which the content of secondary metabolites in hairy roots is at the level or higher than in untransformed tissues.

Species	Analyzed Metabolites	Metabolites Content in Hairy Roots	Metabolites in Plants	Metabolites in Callus Suspension	Reference
*Atropa belladonna*	Total alkaloids contents	1.1–8 mg/gDW	Intact roots—0.3 mg/g DW		[78]
*Atropa belladonna*	Total alkaloids contents	1.32%	In untransformed roots—0.8%		[79]
*Artemisia dubui*	Artemisin	0.603–0.753%.	In untransformed roots—0.001%		[80]
*Artemisia *sp.	Artemisinin and its co-products	1.02 mg/g DW	In untransformed roots—up to 0.687 mg/g DW		[81]
*Panax ginseng*	Ginsenoside	Total content 5.44 mg/g DW	Total content in untransformed roots—4.55 mg/g DW		[82]
*Panax ginseng*	Saponins	2–2.4 times higher compared with native root			[83]
*Panax ginseng*	Ginsenoside	2.88% of dry weight when cultured in 1/8 MS medium for 8 weeks	2.56% of dry weight (cultivated roots were 5 years old)		[84]
*Rubia yunnanensis*	Rubiaceae-type cyclopeptides (RAs)	Amount of (RAs) in 1/2 MS liquid medium—4.611 μg/g DW	In plants in vitro—0.331 μg/g and 4.096 μg/g DW for shoots and roots, respectively. Amount of RAs in seed-borne plants—80.296 μg/g, quinones—7409 μg/g DW	In calli—1.082 μg/g DW	[85]
*Rubia yunnanensis*	Quinones	5067 μg/g DW	24–132 μg/g DW in plants in vitro; in seed-borne plants 7409 μg/g DW	In calli—338 μg/g DW	[85]
*Rubia cordifolia Linn*	Total phenolic contents	139.7 mg/g DW	41.02 mg/g DW of field grown roots	-	[86]
*Rubia cordifolia Linn*	Alizarin	In 5.16-fold than normal roots of field grown plants	5.16 times lower in the roots of the field-grown plants compared with the hairy roots		[86]
*Withania sominifera*	Withanolide A	157.4 μg/g DW	57.9 μg/g DW	-	[87]

## 3. The Collection of Hairy Roots of the Institute of Plant Physiology 

In Russia, the technology of obtaining hairy roots is associated with the name of Kuzovkina I.N. The first object was the hairy roots of *Peganum harmala* L. obtained in 1987 [88,89] (Table 2). 

**Figure 1:**
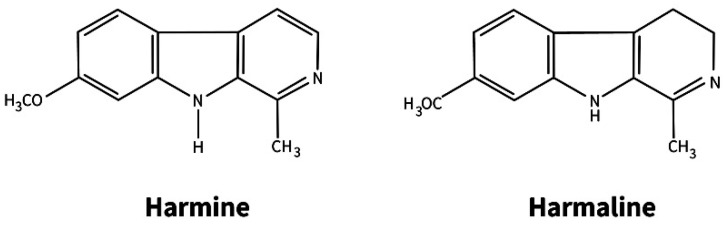
The main β-carboline alkaloids in roots of *Peganum harmala*.

*Peganum harmala* L. (Zygophyllaceae) is a perennial herbaceous plant common in the Mediterranean region of Europe, Central Asia, and southern South America [90,91]. It refers to medicinal plants widely used in folk and traditional Chinese medicine for the treatment of various human diseases [90,91,92,93,94]. The therapeutic effect of extracts from seeds and vegetative parts of the plant is explained by the presence of two classes of alkaloids—quinazoline and indole β-carboline type. Alkaloids of the first class (peganine and its derivatives) are found only in the aerial part, and β-carboline (the main ones are harmine and harmaline) are found only in the roots (Figure 1).

According to the literature, *Peganum harmala* has antibacterial [95], anti-inflammatory [96], anti-fungal [97], and antitumor effects [92], and is used to treat hypertension [98], cough [99], diabetes [100], jaundice [94], malaria [101], tremor paralysis, Parkinson’s disease, and Alzheimer’s disease [92,102]. Despite the wide spectrum of action, the medicine uses peganin hydrochloride (ampoules and tablets) for the treatment of myopathy and myasthenia gravis and harmine hydrochloride for the treatment of encephalitis, tremor paralysis, and Parkinson’s disease [103].

**Figure 2:**
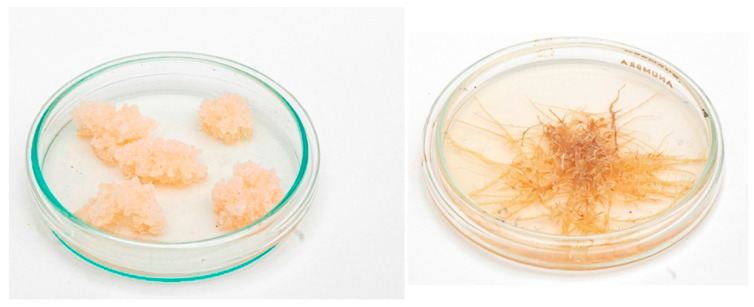
Calli (**left**) and hairy roots (**right**) of *Peganum harmala* obtained in 1987. The photos were taken in 2021.

It was shown by our group that the content of harmine and harmalol (β-carboline alkaloids) in the transformed roots was 30 and 4.3 times higher than in callus, respectively, which indicates the promise of the obtained roots [89]. Calli and hairy roots of *P. harmala* are still maintained in the collection of the IPP RAS (Figure 2). Because the content of harmine in the hairy roots was predominant and only slightly inferior to its content in the roots of intact plants, the resulting hairy roots of *Peganum harmala* can be a source for obtaining an important group of pharmacologically valuable alkaloids which include harmine.

**Figure 3:**
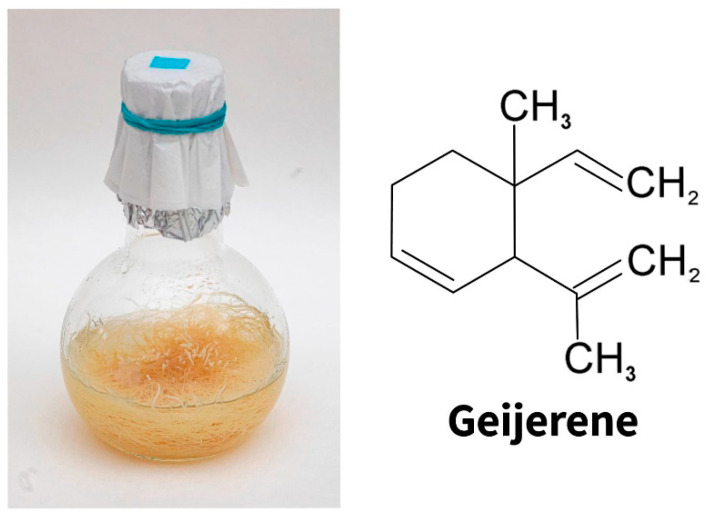
The hairy roots of *Ruta graveolens* (**left**). The main component of the essential oil of common rue is geijarene (**right**).

After, hairy roots of *Ruta graveolens* L. (Rutaceae) were obtained (Figure 3). *Ruta graveolens* (common rue, rue) is a plant with a very rich composition of secondary metabolites (about 200) belonging to various groups of low molecular weight compounds [104]. *Ruta graveolens* contains coumarins, alkaloids, volatile oils, flavonoids, and phenolic acids [104]. Rue has been known as a medicinal plant since ancient times. It has already been used to treat various diseases since the time of Hippocrates [105]. There are numerous reports on the use of rue herbs in the folk medicine of various countries, namely, in Indian and Chinese medicine. Rue has been used for a long time as an analgesic, to eliminate eye problems, and to improve the condition of patients with rheumatism and dermatitis [104,106]. In Russian folk medicine, it was used for heart diseases, disorders of the nervous system, and as an effective abortifacient [107]. Currently, the antiviral, antibacterial, and fungicidal effects of rue have been proven [106]. Along with pronounced pharmacological properties, rue is also used as an essential oil plant. Rue essential oil finds application in the perfumery and food industry [108].

**Figure 4:**
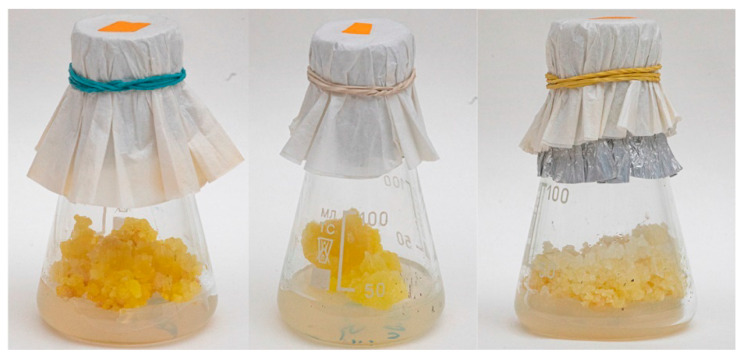
The examples of *Ruta graveolens* calli obtained during the period 1969–1976. The photos were taken in 2021.

The peculiarity of this plant is that the synthesis of these substances is organ-specific, i.e., the root and aerial parts of the plant form various secondary metabolites, and the main part of them is characterized by a kind of fluorescence. With in vitro cultures at its disposal, it is possible to study the spatial distribution of metabolites. From 1969 to 1976, calli of obtained *Ruta graveolens* were obtained by Kuzovkina (Figure 4) to study the biogenesis of furocoumarins and acridone alkaloids. However, it became possible to study the spatial organization of low molecular weight metabolites only with the production of hairy roots of *Ruta graveolens* in 1991 [109,110]. 

Intensively branching genetically transformed roots forming a large number of root apexes turned out to be a convenient object for studying the function of the so-called border cells, which are root cap cells that separate from the root tip during its growth. They are the first to come into direct contact with various representatives of the rhizosphere. Hairy roots of common rue retain the ability to synthesize the essential oil typical of intact plant roots, the main component of which is sesquiterpene geijerene [110]. Interestingly, geijerene (Figure 3) belongs to the number of root-specific volatile metabolites that attract entomopathogenic nematodes eating the larvae of a dangerous root pest of young citrus plants (*Rutaceae*), the weevil *Diaprepes abbreviate* [111]. 

Thus, hairy roots can be a model not only for the spatial study of secondary metabolites but also for the study of allelopathic relationships between roots and soil micro- and macrobiota.

When creating the collection, the main attention was paid to those plants in which the roots were found to contain low molecular weight metabolites that are of practical interest and are used in the medical and food industries. For this purpose, hairy roots of *Glycyrrhiza uralensis*, *Rauwolfia serpentina, Rubia tinctorum*, *Rhodiola rosea*, various species of the genus *Scutellaria*, and other most important medicinal plants were obtained [112].

**Figure 5:**
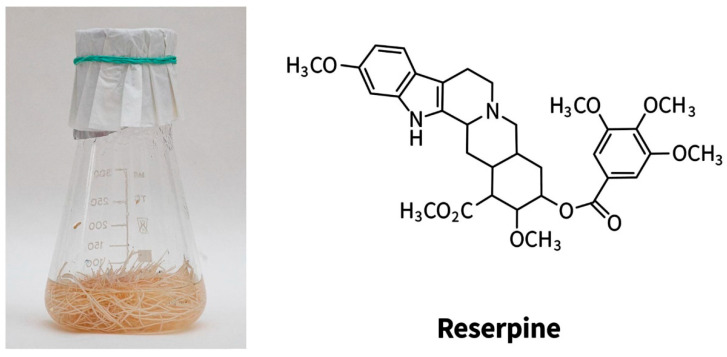
The hairy roots of *Rauwolfia serpentina* (**left**) and the main alkaloid (**right**).

*Rauwolfia serpentina* Benth. (Apocynaceae) has pronounced pharmacological properties. The main use of *R. serpentina* was for snake and insect bites, fever, cholera, diarrhea, as a mild sedative for children, and in Java, it was used as an anthelmintic [113,114,115]. To date, it has been shown that *Rauwolfia serpentina* has antibacterial [116], antifungal [117], anti-inflammatory [118], antidiabetic, mosquito larvicidal, antihistamine, antidiarrheal [119], hypoglycemic and hypolipidemic, anticancer, as well as sedative [120] and hepatoprotective activities [113,121]. Currently, more than 80 alkaloids have been isolated from this plant [122]. However, reserpine is a pharmacologically more potent alkaloid [122]. On the basis of *Rauwolfia serpentina* alkaloids, the drug “Reserpine” against arterial hypertension and “Ajmalin”—an antiarrhythmic agent was released. The hairy roots of *Rauwolfia serpentina* were obtained in 1990 and are cultivated to the present (Figure 5).

*Rubia tinctorum* L. (Rubiaceae) is also a medicinal plant, according to modern studies, containing about 250 compounds with different chemical classes [123]. The main components are anthraquinones and their derivatives such as alizarin and purpurin, which have a diuretic, antispasmodic, and laxative effect [124]. At present, the antitumor, hepatoprotective and antidiabetic properties of the roots of common madder have been shown [123,125,126]. Besides that, the roots of common madder have been used as a dye for dyeing fabrics and applying patterns since ancient times [127,128].

**Figure 6:**
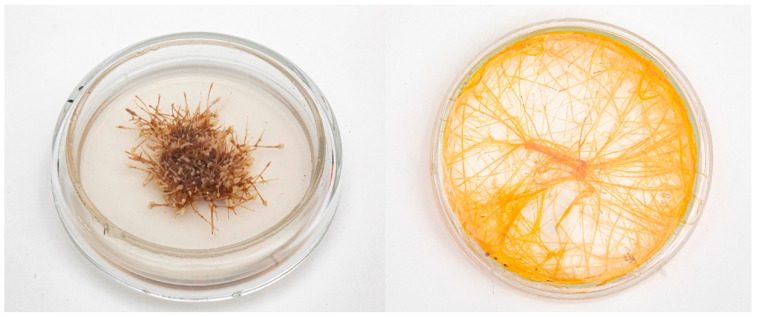
Hairy roots of *Glycyrrhiza uralensis* (**left**) and *Rubia tinctorum* (**right**). The photo was taken in 2021.

The hairy roots of *Rubia tinctorum* were obtained in 1991 and are cultivated to the present in the collection of hairy roots of the Institute of Plant Physiology (Figure 6).

*Glycyrrhiza uralensis* (licorice) (Fabaceae) is a plant that has found its application both in the food and pharmaceutical industries. Licorice contains many different substances, among which glycyrrhizin and glycyrrhetic acid can be considered the main ones. The plant has been known since ancient times. Licorice was considered a panacea for many diseases in oriental medicine. The Egyptians, Greeks, and Romans recommended licorice as a remedy to help fight physical stress and fatigue [129]. In our country (Russia), licorice has always been the largest harvesting object, as well as the subject of raw material exports. Biologically active constituents of licorice have antiviral and anti-inflammatory effects; it has recently been shown that it can also be used to treat alcoholic liver damage [130,131,132]. Licorice root is also used in the food industry as a sweetener, flavor, and aroma enhancer [129,133].

The hairy roots of *Glycyrrhiza uralensis* were obtained in 1990 and are cultivated to the present in the collection of hairy roots of the Institute of Plant Physiology (Figure 6).

**Figure 7:**
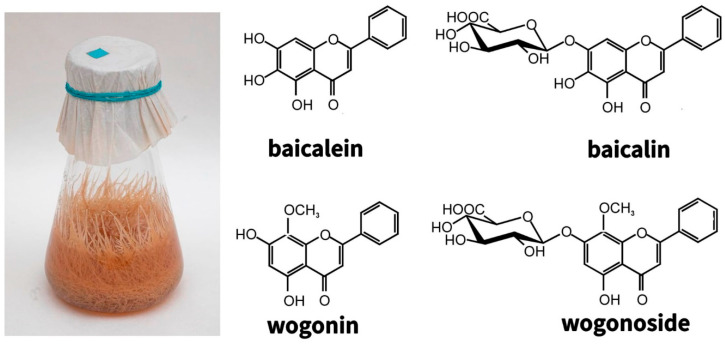
Hairy roots of *Scutellaria baicalensis* obtained in 1996 (**left**). The main root-specific flavones (**right**). The photo was taken in 2021.

The genetic stability of hairy roots and their ability to maintain the synthesis of low molecular weight metabolites under in vitro conditions at the level of the roots of the whole plant formed the basis for studies conducted with the roots of valuable medicinal plants of the genus *Scutellaria*. The roots of Baikal skullcap, which have been steadily growing for more than 25 years, synthesize flavones typical of the roots of this plant—glucuronides: baicalin and wogonoside, and aglycones: baicalein and wogonin [134,135,136,137,138] (Figure 7).

Although the content of major metabolites in hairy roots is three times lower than in the roots of intact plants, this disadvantage is compensated by the rapid growth and year-round cultivation of hairy roots. However, if the dominant metabolite of the *Scutellaria baicalensis* roots of intact plants is the glucuronide baicalin and, accordingly, its aglycone baicalein, then the main flavone in hairy roots is the mono-O-methylated glucuronide wogonoside and its aglycone wogonin [134,135,138]. Later it was suggested that the increase in the content of monomethylated flavones is associated with the climatic features of the region (Dauria, Russia), where the plants were taken for transformation [139]. Nevertheless, when obtaining other members of the genus *Scutellaria (Scutellaria andrachnoides* (together with Kyrgyz colleagues), *Scutellaria przewalskii, Scutellaria pycnoclada,* and *Scutellaria lateriflora* taken from different climatic zones, increased content of mono-O-methylated flavones was also shown [136,140]. This suggests more subtle regulatory mechanisms that are currently being investigated. The practical significance of such a ratio of flavones in skullcap hairy roots was assessed after the publication of Japanese researchers in 2009, who showed that the aglycone wogonin selectively induces apoptosis only in cancer cells, while not affecting normal cells [141]. Its antitumor activity was confirmed by in vivo studies, which opened up the possibility of the clinical application of wogonin [142,143,144]. Wogonin has been confirmed to be effective against neurodegenerative diseases, including Alzheimer’s disease [145]. The anti-coronavirus properties of wogonin have also been recently discovered [146,147,148]. Because the aglycone wogonin has a pronounced therapeutic effect, but the main methylated flavone in the hairy roots of *S. baicalensis* obtained by us is wogonoside glucuronide, the latter must be hydrolyzed. Some publications propose different ways of obtaining aglycones from glucuronides, one is hydrolysis using β-glucuronidase from various microorganisms. For instance, 90% of glucuronides were hydrolyzed to aglycones within 3 h with the help of β-glucuronidase from *Lactobacillus delbruecki* [149]. At the same time, it is known that the Baikal skullcap contains its own β-glucuronidase (sGUS) [150,151]. We have conducted a number of studies of the relationship between the content of basic flavones and the activity of the sGUS both in the maintained *S. baicalensis* hairy roots strain and in the undifferentiated callus and suspension cultures obtained from it [26,77]. As a result, it was shown that the activity of sGUS during the cultivation cycle of *S. baicalensis* hairy roots correlated with the content of wogonin more than with the content of baicalein. We have also demonstrated that the content of wogonin increases in response to mechanical stress and is presumably associated with the protection of plants from biotic stress, in particular, from insect pests. It should be noted that undifferentiated cultures (calli and suspensions) of *S. baicalensis* had a different ratio of flavones with a predominance of the baicalin/baicalein pair, in contrast to hairy roots. Interestingly, sGUS activity in hairy roots was 10 times higher than in undifferentiated cultures [137]. In the future, we plan to study the possibilities of sGUS activation and an increase in the level of biologically active flavones-aglycones.

Thus, in our studies conducted on the hairy roots of members of the genus *Scutellaria* from the collection of hairy roots of the Institute of Plant Physiology, it was shown that they contain the same set of flavones as the intact roots. However, the roots of intact plants and hairy roots differ in the ratio of flavones.

This makes our objects unique both for research and commercial use.

Another rare medicinal plant from which both hairy roots and undifferentiated cells were obtained was *Rhodiola quadrifida* Pall. (Crassulaceae), known for its medicinal properties [152,153,154]. It belongs to the alpine species and its range is rapidly shrinking [155]. It should be pointed out that the content of the main metabolites (salidroside and rosavin) was higher in undifferentiated callus cultures than in hairy roots [156]. The results are of considerable interest for further research.

At present, the collection of hairy roots includes 38 hairy roots strains belonging to 25 plant species and 16 lines of callus cultures (Table 2).

**Figure 8:**
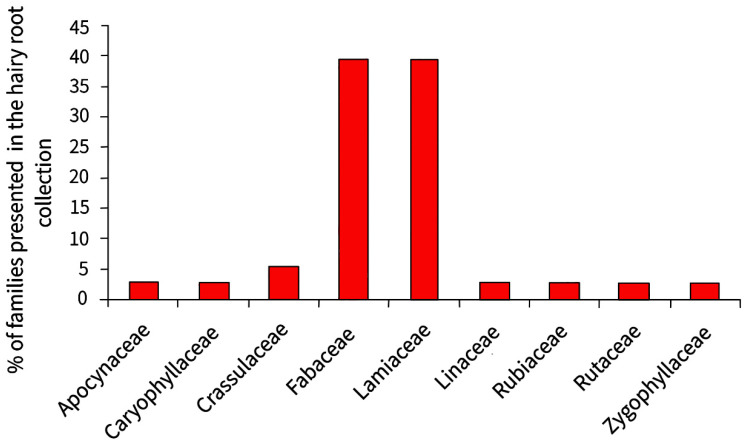
The percentage of plant families represented in the collection of hairy roots.

The collection mainly maintains hairy roots of medicinal plant species (84%), and the two families that are predominantly represented are *Fabaceae* and *Lamiaceae* (Figure 8).

**Table 2:** Some strains in the hairy roots collection of Institute of Plant Physiology.

Family	Species Name	Number of Lines	Type of the Culture	The Year Obtained	Origin of the Culture	Secondary Metabolites	Note	Possible Use	Literature References in Which This Strain Is Mentioned
Apocynaceae	*Rauwolfia serpentina* L. (Benth.)	1	Hairy roots	1990	Leaves of juvenile plants	Contain indole alkaloids (vomilenine, vinorine, perakine) higher than in suspension culture.	The total alkaloid content was 3 times lower compared with the suspension culture. There is no raucaffricine alkaloid, which predominates in suspension culture.	Pharmacology, medicine	[112]
Caryophyllaceae	*Silene vulgaris* L.	1	Hairy roots	2002	-	-	-	Phytoremediation (accumulation of heavy metals)	-
Crassulaceae	*Rhodiola quadrifida Pall*	2	Hairy roots	2017	Cotyledons and hypocotyls	Contain salidroside, rosavin	Tyrosol and rosarin are missing. The content is significantly lower than in callus tissue.	Pharmacology, preserving of rare and endangered species	[156]
	*Rhodiola quadrifida Pall*	4	Calli	2019	Hairy roots	Contain salidroside, rosavin, rosin	Thyrosol and rosarin are absent		
Fabaceae	*Hedysarum* sp.	5	Hairy roots	2001–2019	Juvenile seedlings	Isoflavones (ononine)	-	Pharmacology, medicine	[157]
	*Glycyrrhiza uralensis* L.	1	Hairy roots	1990	Hypocotyle of juvenile plant	Phenolic derivatives	-	Pharmacology, medicine	-
	*Lupinus polyphyllus* L.	1	Hairy roots	1990	Hypocotyle of juvenile plant	Isoflavone glycoside		Biotechnology, feed additives	
	*Ononis* sp. L.	4	Hairy roots	1993–1994	Hypocotyle of juvenile plant	isoflavonoids			
	*Sophora korolkovii Koehne*	1	Hairy roots	2004	Hypocotyle of juvenile plant	Phenolic compounds			
	*Trifolium repens* L.	1	Hairy roots	1991	Hypocotyle of juvenile plant	A model object for studying arbuscular mycorrhizae			
	*Medicago sativa* L.	2	Hairy roots	2013	Hypocotyle of juvenile plant	Phytoremediation			
Lamiaceae	*Scutellaria baicalensis Georgi*	3	Hairy roots	1993, 2018, 2021	Leaves of juvenile plant	Contains flavones (baicalin, baicalein, wogonin, wogonoside)	The content of methylated flavones is higher than in the roots of intact plants	Pharmacology, medicine, food industry	[135,136,137,138,158]
	*Scutellaria baicalensis Georgi*	2	Calli		Hairy roots	Contains flavones (baicalin, baicalein, wogonin, wogonoside)	The main flavones are baicalin and baicalein, as in the roots of intact plants.		
	*Scutellaria androchnoides*	1	Hairy roots	2006	Cotyledon and hypocotyl	The dominant compounds are acteoside (phenylethanoids) and the four main flavones of representatives of *Scutellaria* sp.: baicalin, wogonoside, wogonin and baicalein	The content of acteoside is 10 times higher, and the content of methylated flavone wogonoside is 3 times higher compared with the roots of intact plants	Pharmacology, medicine	[140]
	*Scutellaria androchnoides*	1	Calli	2006	Hairy roots	The dominant metabolites are acteoside and the methylated flavones wogonoside and wogonin	The content of acteoside is 2.5 times, wogonoside 1.5 times higher than in the roots of intact plants	Pharmacology, medicine	
	*Scutellaria przewalskii*	1	Hairy roots	2014, 2020	Cotyledons, hypocotyls of sterile-grown plants	Based on HPLC data, 17 flavones were found, among which the main metabolites are baicalin and wogonoside glucuronides	The content of the main metabolites is higher than in all of the above representatives of *Scutellaria*	Pharmacology, medicine	[136]
	*Scutellaria lateriflora*	2	Hairy roots	2020	Hypocotyls of sterile-grown plants	Contains flavones (baicalin, baicalein, wogonin, wogonoside)	The content of flavones is 4.57 mg/g DW, that is lower than in all the species of hairy roots studied by us	Pharmacology, medicine	[136]
	*Scutellaria pycnoclada*	8	Hairy roots	2020	Hypocotyls of sterile-grown plants	In contrast to other lines of skullcaps, the ratio of main flavones is close to that of the roots of intact plants		Pharmacology, medicine	[136]
Linaceae	*Linum usitatissimum* L.	1	Hairy roots	1995	Cotyledons of sterile-grown seedlings	Cyanogenic glycosides, lignan		Pharmacology, medicine	
Rubiaceae	*Rubia tictorum* L.	1	Hairy roots	1991		Anthraquinones	The content of anthraquinones is 2.5% by dry weight. In the roots and rhizomes of an intact plant—5.2%	Pharmacology, medicine	[159]
Rutaceae	*Ruta graveolens* L.	1	Hairy roots	1991		Hypocotyle of a juvenile plant	Acridon alkaloids	Study of the spatial distribution of acridone alkaloids	[109]
	*Ruta graveolens* L.	8	Calli	1969, 1970, 1978, 1980, 1999	Stem of whole plants, hypocotyle of juvenile seedlings, roots of juvenile seedling	Acridonalkaloids		Study of the biosynthesis of acridone alkaloids	[160,161]
Zygophyllaceae	*Peganum harmala* L.	1	Hairy roots	1988	Stem of a juvenile plant	β-carbolinealkaloids (harmine, harmalol, harmaline), serotonin		Study of the distribution of secondary metabolites in plant roots. Pharmacology	[52]
	*Peganum harmala* L.	1	Calli	1988	Spontaneous callus formation on a juvenile plant stem	β-carboline alkaloids, serotonin			

The collection contains 14 genera of plants of the *Fabaceae* family including both forage plants (*Lupinus polyphyllus* L., *Medicago sativa* L., and *Trifolium repens*) and medicinal plants (*Sophora korolkovii* Koehne, *Glycyrrhiza uralensis* L., *Ononis* sp., and *Hedysarum* sp.). The *Lamiaceae* family is only represented in the collection by the genus *Scutellaria*, which is widely known for its medicinal properties. The number of lines obtained from each species of the genus *Scutellaria* is from 1 to 8, so the total number of lines of hairy roots belonging to the *Lamiaceae* family is equal to the number of lines of the *Fabaceae* family. The remaining families are represented by single species, each of which is maintained by one line of hairy roots.

**Figure 9:**
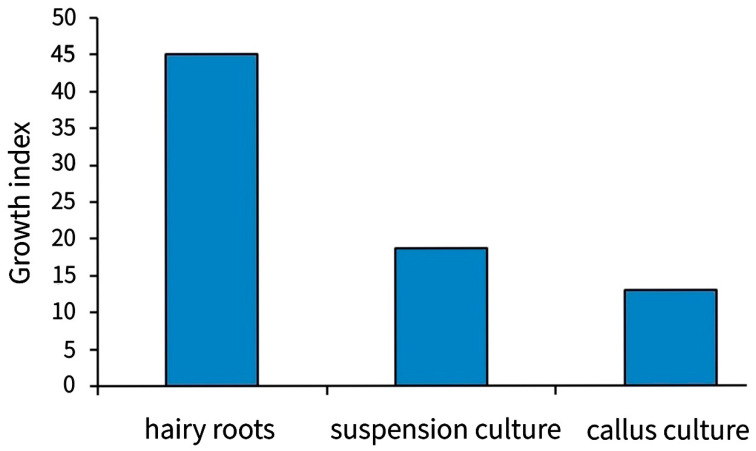
Growth index of the *Scutellaria baicalensis* Georgi in in vitro cultures.

For comparative studies, undifferentiated callus cultures are maintained in the collection (Table 2), however, they grow much slower than hairy roots (Figure 9).

The hairy roots are continuously cultivated in our collection. Surely, the permanent cultivation of hairy roots is laborious, but cryopreservation of hairy roots is not an easy task, requiring the development of an individual protocol for each line of hairy root cultures [26]. Nevertheless, due to the genetic stability of hairy roots, permanent transplants do not affect the level of synthesis of metabolites.

It should be noted that the presence of the collection makes it possible to conduct a wide range of studies that cannot be done with one or more lines at one’s disposal (Table 2). The results obtained can be used in practical activities for the production of biologically active substances in the pharmaceutical industry. The use of hairy roots is becoming increasingly important due to the reduction of the areas of medicinal plants and the production of new strains of hairy roots.

## 4. Conclusions

Hairy roots are a unique in vitro system capable of rapid growth on hormone-free media and significant synthesis of secondary metabolites characteristic of both underground and aboveground parts of plants. Since they are differentiated structures, they can be used as a model to study the spatial distribution of secondary metabolites in plant roots. The collection of hairy roots can be not only a tool for conducting various fundamental and applied research but also a way to preserve rare and endangered species. It is also extremely interesting to reveal the features of the synthesis of secondary metabolites in differentiated and undifferentiated cultures obtained from them, as well as on media of different compositions. The latter can be of great practical importance. The prospects for the development of the collection of hairy roots include the expansion of the range of plant families, from which they will be obtained, including at the expense of “stable” monocotyledonous species.

## Data Availability

The data presented in this study are available on request from the corresponding author.
